# Mitigation of quorum sensing mediated virulence factors of *Pseudomonas aeruginosa*: the role of Meldrum’s acid activated furan

**DOI:** 10.3389/fmicb.2023.1272240

**Published:** 2024-01-03

**Authors:** Ajmal Sadik, Jithin P. Viswaswar, Ambili Rajamoney, Anjali Rekha, Darsana M. Raj, Deepthi Prakashan, Mydhili Vasudevan, J. S. Visakh, Dhannya Renuka, Sreetha Hely, Sanu Korumadathil Shaji, Prakash R. Chandran, Geetha Kumar, Sobha Vijayan Nair, Jayalekshmi Haripriyan

**Affiliations:** ^1^School of Biotechnology, Amrita Vishwa Vidyapeetham, Amritapuri, Kerala, India; ^2^Department of Chemistry, Mannam Memorial N.S.S. College, Kottiyam, Kerala, India

**Keywords:** activated furan, *Chromobacterium violaceum*, *Pseudomonas aeruginosa*, quorum sensing, virulence, antimicrobial resistance, biomass

## Abstract

The rapid emergence of drug resistant pathogens is a major threat which has warranted the development of alternative strategies to combat infectious diseases. In this work, we have tested the anti-virulent activity of Meldrum’s acid activated furan (MAF) and 1,3-dimethyl barbituric acid activated furan (BAF) against *Chromobacterium violaceum* and *Pseudomonas aeruginosa*. It was found that MAF significantly reduced the violacein production and biofilm formation of *C. violaceum* at sub-inhibitory concentrations. The quorum sensing (QS) regulated virulence factors of *P. aeruginosa* including biofilm formation, motility, pigment production, and elastase activity were also found to be reduced considerably at sub-inhibitory concentrations of MAF. Additionally, MAF downregulated the expression of genes in the QS circuitry of *P. aeruginosa*, demonstrating the potential of MAF in lowering the pathogenicity of *P. aeruginosa*. *In silico* studies demonstrated the potential of MAF to compete with the signaling molecules of *C. violaceum* and *P. aeruginosa* for the QS receptor interaction. *In vivo* studies using *Caenorhabditis elegans* demonstrated the anti-pathogenicity of MAF by enhancing the survival of *P. aeruginosa*-infected *C. elegans*. These results suggest that activated furan compounds could be potential inhibitors of QS-mediated virulence factors in *C. violaceum* and *P. aeruginosa*, encouraging their use in combating multidrug-resistant pathogens.

## 1 Introduction

Over the years, antimicrobial resistance has become a global threat that affects both high-income countries (HICs) as well as low- and medium-income countries (LMICs). Hence, there is considerable interest in the pharmaceutical industry towards employing innovative strategies in the development of novel drug candidates. Biomass-derived feedstock-based drug discovery is one among the most promising strategies, which could lead to a wide spectrum of potential drug candidates via sustainable means. Valorization of biomass-derived feedstock has been successfully employed as a sustainable strategy for manufacturing commodity chemicals (Tuck et al., 2012).

For instance, furfural and hydroxymethyl furfural which are derived from lignocellulosic biomass are employed as building blocks for tetrahydrofuran, dimethylfuran, ethyl levulinate, etc. (Dutta et al., 2012). However, our survey of the literature revealed that there is a paucity of reports on the application of this approach in drug discovery. This study is focused on the efficacy of activated furan compounds, Meldrum’s acid activated furan (MAF) and 1,3-dimethyl barbituric acid activated furan (BAF) as anti-virulent agents. Recently we reported MAF as an effective agent for protein quantification (Ajayan et al., 2023).

Microorganisms utilize cell-to-cell communication mechanisms to interact within a population known as quorum sensing (QS) to promote collective behaviors. When the bacterial population attains a critical threshold, QS regulates the expression of virulence genes (Mukherjee and Bassler, 2019). The synthesis of small, diffusible, signal molecules called N-acyl homoserine lactones (AHLs), that travel in and out of bacterial cells, bind to receptor molecules (LuxR), which in turn activates the formation of dimers or multimers of the LuxR-AHL complex. These function as transcriptional regulators of target genes of the QS systems. To regulate the physiological activities including the virulence factors such as biofilm formation, motility, sporulation, bioluminescence, and the exchange of DNA, bacteria exploit QS communication circuits (Zhou et al., 2020). Therefore, QS is a signaling mechanism that enables them to function as multicellular organism. Bacterial pathogens use QS to regulate the production of virulence factors and make the QS system a target for decreasing pathogenicity (Martínez et al., 2019). Thus, QS inhibitors or blockers may be of vast importance for treating bacterial infections (Jiang et al., 2019).

*Chromobacterium violaceum* and *Pseudomonas aeruginosa* are gram negative bacteria that have a wide geographic distribution. *C. violaceum* forms biofilm and produces the pigment violacein in response to QS-regulated gene expression, while *P. aeruginosa* is a major nosocomial pathogen that leads to a wide variety of diseases, the virulence characteristics of which are regulated by quorum sensing (Asfour, 2018). Thus, in this work, we have explored the anti-QS and anti-virulent properties of Meldrum’s acid activated furan (MAF) and 1,3-dimethyl barbituric acid activated furan (BAF) against *C. violaceum* and *P. aeruginosa*.

## 2 Materials and methods

### 2.1 Materials

All the strains used in this study, *Chromobacterium violaceum* (ATCC 12472) and *P. aeruginosa* PAO1 (ATCC 15692), were maintained in glycerol (80%) and stored at −80°C. For every new experiment, fresh stocks were subcultured. The wild-type strain of *Caenorhabditis elegans* (Bristol N2) was used for the *in vivo* experiments. Furanone C-30 (CAS No.: 247167-54-0, product No.: 53796) was purchased from Merck, India. Analytical grade chemicals and solvents were used for the synthesis of activated furans. Furfuraldehyde (CAS No.: 98–01-1, Item No.: 0106146), Meldrum’s acid (CAS No.: 2033-24-1, Item No.: 0113207), 1,3-dimethyl barbituric acid (CAS No.: 769–42-6 Item No.: 0104393), and dichloromethane (CAS No.: 75–09-2, Item No.: 0113271) were purchased from Spectrochem, India. Sodium bisulphite (CAS No.: 7631-90-5, MB358), sodium bicarbonate (CAS No.: 144–55-8, PCT1535), and sodium sulfate (CAS No.: 7757-82-6, GRM6425) were purchased from HiMedia, India.

### 2.2 Synthesis of activated furan compounds

**Figure 1:**
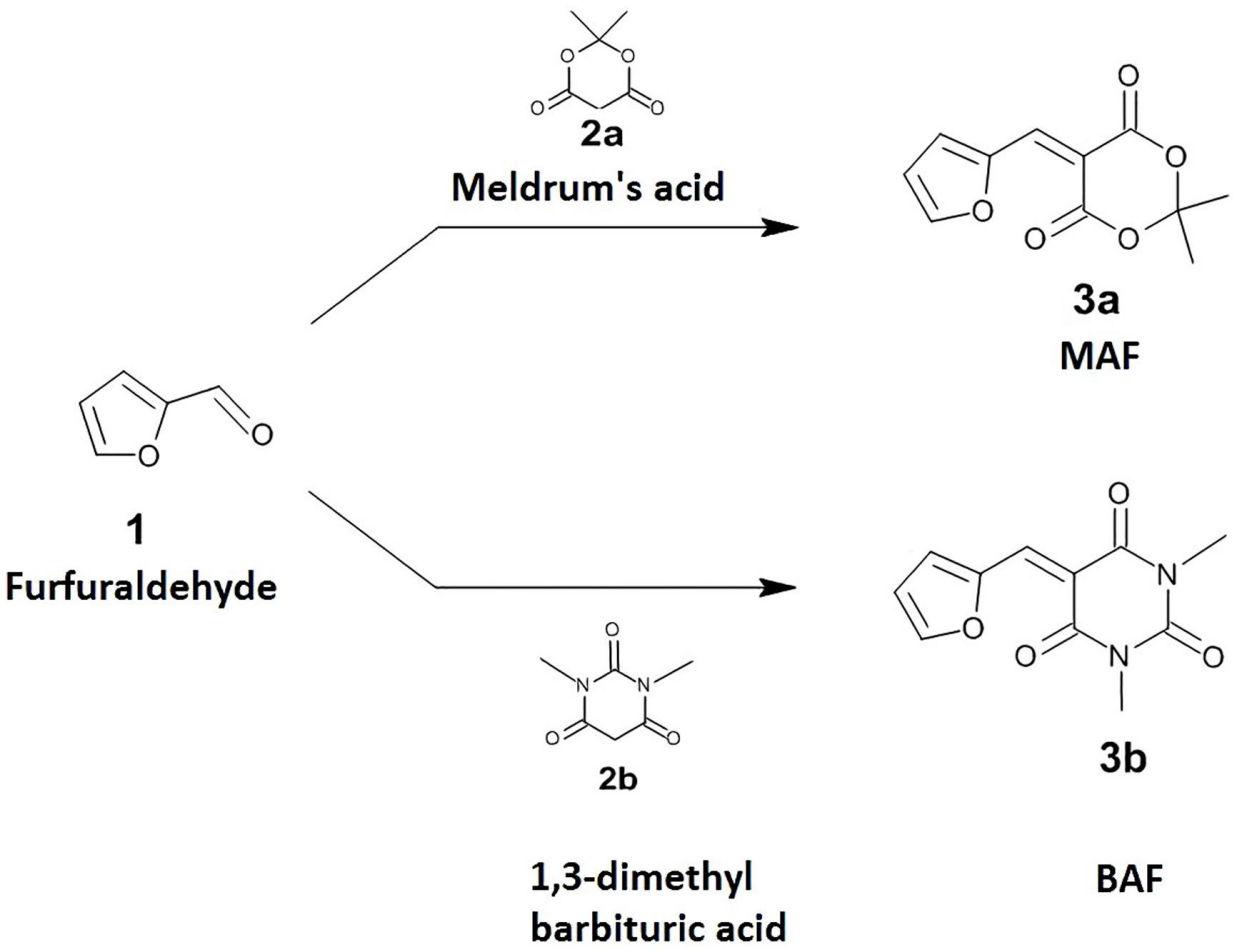
Synthesis of 5-(furan-2-yl methylene)-2,2-dimethyl-1,3-dioxane-4,6-dione (MAF,3a) and 5-(furan-2-ylmethylidene)-1,3-dimethyl-1,3-diazinane-2,4,6-trione (BAF,3b).

Both Meldrum’s acid activated furan (MAF-**3a**) and 1,3-dimethyl barbituric acid activated furan (BAF-**3b**) were prepared according to the reported procedure (Helmy et al., 2014). Thus, Meldrum’s acid-**2a** (1.5 g, 10.4 mmol) or 1,3-dimethyl Barbituric acid-**2b** (1.5 g, 9.6 mmol) and furfuraldehyde-**1** (961 mg, 10 mmol) were added to 30 ml water in a 100 ml round bottom flask and the resulting heterogeneous mixture was heated to 75°C with stirring for about 3 h. The mixture was then cooled to room temperature and the product was extracted using dichloromethane. It was then washed sequentially with 50 ml saturated aqueous NaHSO_3_ and then finally with 50 ml of brine. The organic layer was dried over anhydrous sodium sulfate and the solvent was removed using rotary evaporation to yield **3a-b** as a bright yellow crystalline powder in 60–75% yields (Figure 1). Purified compounds were characterized by 1HNMR and 13CNMR spectroscopy and mass spectrometry (Supplementary Figures S1A–F).

### 2.3 Determination of minimum inhibitory concentration (MIC)

MIC was determined by the broth microdilution method. Briefly, overnight microbial cultures were diluted in Luria broth (LB, Himedia, India) to obtain 1 × 10^6^ CFU/ml. Diluted cultures grown with different concentrations of MAF and BAF ranging from 100 to 1,000 μg/ml were used for the study. The MIC and sub-MIC were determined by measuring the absorbance after incubation at 37°C for 24 h (Kowalska-Krochmal and Dudek-Wicher, 2021).

### 2.4 Detection of anti-quorum sensing activity in *Chromobacterium violaceum*

Violacein production by *C. violaceum* in the presence of test compounds was evaluated by extracting and quantifying the violacein as described earlier (Lu et al., 2022). *C. violaceum* was grown at 37°C for 24 h. Approximately 1 ml of bacterial culture was centrifuged at 12,000 rpm for 10 min, the supernatant was aspirated off, and 1 ml DMSO was added to the pellet. This was vortexed vigorously for 30 s to solubilize the violacein and centrifuged at 12,000 rpm for 10 min. Violacein containing supernatants (100 µl) were added to microtiter plates and the absorbance read at a wavelength of 585 nm.

### 2.5 Bacterial growth curve analysis

The effect of MAF (100, 200, 400 µg/ml) on bacterial growth was studied using the method of Peerzada et al. (2022). Various concentrations of MAF were added to *P. aeruginosa*-containing LB broth in 96 well plates to reach a final volume of 200 μl. Every 1 h, the cell density was evaluated at 600 nm using a Synergy BioTek microplate reader. The growth curve was plotted by taking the average reduction in Optical Density (OD) of three measurements.

### 2.6 Motility phenotypes

#### 2.6.1 Swarming motility

Test compounds at sub-MIC concentration were added in media comprising agar (0.5%), peptone (0.5%), yeast extract (0.2%), and glucose (1%) and were used for bacterial growth. Plates were point-inoculated at the center of the agar plates with an overnight culture of *P. aeruginosa* PAO1 and incubated for 16 h at 37°C (Cai et al., 2020).

#### 2.6.2 Swimming and twitching motility

Swimming and twitching motility was determined as reported by Cai et al. (2020). The bacterial culture was spot inoculated in the center of the media (1% tryptone, 0.5% NaCl, and 0.3% agar) and incubated for 16 h at 37°C. The circular zone measured from the point of inoculation which was developed due to bacterial migration was used to assess the swimming motility. Twitching motility was determined after 24 h incubation of cells at 37°C, which were stab inoculated in 1% LB agar.

### 2.7 Effects of test compounds on biofilm formation

Bacteria were grown at 37°C for 18–24 h, diluted to an OD of 0.1 (1 × 10^8^ CFU/ml) at 600 nm, which were then added with test compounds and incubated for 24 h at 37°C. Following incubation, the planktonic phase cultures were removed from the wells, washed thoroughly with water, and allowed to air dry. Bacterial cells adhered on biofilm were stained with 0.1% (w/v) crystal violet (aq.) for 5 min and washed again with water to remove the unbound dye. After the wells were allowed to dry, the biofilm bound dye was eluted using 33% glacial acetic acid for 5 min, and the absorbance was measured at 600 nm (Adonizio et al., 2008). Furanone C-30, a known anti-biofilm agent of *P. aeruginosa*, was used as positive control (Muñoz-Cázares et al., 1847).

### 2.8 Assessment of pigment production

Following the reports of Liang et al., pyocyanin was extracted and measured (Liang et al., 2011). Chloroform (3 ml) was added to the culture supernatant, vortexed for 2 min, and centrifuged at 8,000 rpm for 5 min to extract the pyocyanin. Then 1 ml 0.2 M HCl was added to the transferred chloroform layer and subjected to 5 min of centrifugation at 8,000 rpm. After removing the top layer (0.2 M HCl), the pyocyanin concentration was measured as absorbance of the chloroform layer at 520 nm multiplied by 17.072.

### 2.9 Effects of MAF on expression of QS genes

The effect of test compounds on QS gene expression was studied by treating bacterial cultures with varying concentrations of the compounds (Omanakuttan et al., 2016). Pre-chilled vials were used to transfer aliquots (1.5 ml) of bacterial cultures, and the cells were extracted by centrifugation (6,000 g, 10 min) eliminating extracellular membranes, polysaccharides, and DNA, preparing the samples for RNA extraction. The procedure involves the addition of TRIZOL (Cat# ORN012X, Origin, India) reagent to the pellet followed by incubation at room temperature for 3 min which was then added to 0.1 ml ice-cold chloroform and mixed for 15 s. The sample was then incubated for 15 min and centrifuged at 8,000 rpm for 15 min at 4°C. Approximately 0.25 ml of 100% isopropyl alcohol was added to the upper chloroform layer and incubated at −20°C for 30 min. The mixture was centrifuged at 14,000 rpm for 10 min at 4°C to obtain pellets, which were then rinsed with 1 ml of 70% ethanol and centrifuged for an additional 5 min. Using a Nanodrop spectrophotometer (Thermo Scientific), total RNA was measured after dissolving the pellet in nuclease-free water. As directed by the manufacturer (Origin, India), 1 μg of template RNA, the PCR master mix (Cat# ODR31, Origin, India), and 1 pM/μl of specific primers were mixed (Omanakuttan et al., 2016). One-step reverse transcription PCR using an Eppendorf Mastercycler was done for the amplification and detection of the product (Eppendorf, Hamburg, Germany). The difference in the intensity of the bands in agarose gels was quantified with ImageJ software (Lo et al., 2016). Fold change was calculated by comparison with the untreated control. The housekeeping *rpsL* gene was employed as a reference.

### 2.10 Measuring elastase B activity of *Pseudomonas aeruginosa* using elastin–Congo Red assay

The assay used Elastin–Congo Red (ECR, Sigma, E0502) as the substrate to measure the activity of the enzyme (Haripriyan et al., 2018). PAO1 culture supernatant in the presence of MAF was added to 900 μl ECR buffer (100 mM Tris, pH 7.2) containing 1 mM CaCl_2_ and 20 mg ECR. This mixture was then incubated on a shaker incubator for 3–4 h at 37°C. The insoluble ECR was removed by centrifugation at 8,000 rpm and the OD of the supernatant was determined at 495 nm.

### 2.11 *Caenorhabditis elegans* lethality assay

Age-synchronized L4 nematodes were transferred to individual wells (~ 30 nematodes/well) of an OP50-seeded 24-well plate containing various concentrations of MAF (100, 200, and 400 μg/ml). The number of live and dead nematodes was counted after 24 h at 20°C. Worms were scored as dead when physical stimuli (e.g., touching using a small metal wire) failed to generate any response. Experiments were repeated on three different days and the survival curve was plotted (Lanzerstorfer et al., 2021).

### 2.12 Survival assay of *Caenorhabditis elegans*

Survival assay of *C. elegans* was assessed by paralytic killing in liquid media (Kong et al., 2014). *C. elegans* was grown in nematode growth medium (NGM) at 20°C and fed OP50 strain of *Escherichia coli* according to a standard protocol (Brenner et al., 1974). *E. coli* was cultured overnight in Luria–Bertani (LB) medium. The eggs were obtained by treating gravid mature adults with hypochlorite followed by rinsing with M9 buffer and transferred to a microtiter plate with 89% M9 buffer (0.3% KH_2_PO_4_, 0.5% NaCl, 0.6% Na_2_HPO_4_, 1 M MgSO₄, 10% Luria–Bertani (LB) broth, and 1% cholesterol). The synchronized L1 larvae (25–30) were infected with *P. aeruginosa* (10^8^ CFU/ml) which was previously treated with MAF. The control was uninfected *C. elegans* with *E. coli* OP50 and infections were carried out in triplicates. The survival of the worms was assessed at different time points (0, 8, and 24 h). Graph Pad Prism was used to plot the Kaplan and Meier survival curves.

### 2.13 Computational docking studies

To get further insights into the mechanism of action of the compound with different virulence proteins, computational docking studies were performed. The 3D structure of the compound was created using ChemDraw and the crystallographic structures of CViR (PDB ID: 3QP4), LasR (PDB ID: 2UV0), and PqsR (PDB ID: 4JVI) were downloaded from the Protein Data Bank (PDB). Since the crystal structure of RhlR was not available in the PDB, homology modeling of the protein was done using SWISS-MODEL homology modeling server (Waterhouse et al., 2018). The Computed Atlas of Surface Topography of proteins (CASTp) server was used to find the binding pockets in the modeled protein (Tian et al., 2018). Autodock 4.2 was used for the site-directed docking studies. The compounds were docked using a Lamarckian genetic algorithm, with a population size of 300. The maximum number of energy evaluations was set to 25 × 10^6^ and the docking results were visualized in PyMol. The best docking pose of MAF was selected for further analysis. The BIOVIA Discovery Studio Visualizer was used for the visualization of 2D interaction map of ligand protein complexes.

### 2.14 Statistical analysis

All data are presented as the mean ± standard deviation (S.D). Error bars represent variations within the experiment. Analysis was performed using GraphPad Prism 8.0 (GraphPad Software Inc., CA, USA). The values of *p* < 0.05, *p* < 0.01, *p* < 0.001, and were considered as statistically significant (*), very significant (**), and highly significant (***) differences, respectively, unless otherwise mentioned.

## 3 Results

The anti-virulent activity of MAF and BAF, two activated furan derivatives derived from furfural via a facile reaction with Meldrum’s acid and 1,3-dimethyl barbituric acid, respectively, were investigated. Experimental and computational studies prove that these compounds could be potential inhibitors of QS regulated virulence factors in *C. violaceum and P. aeruginosa*. The details of these studies are given below.

### 3.1 Minimum inhibitory concentration (MIC) of test compounds

The broth micro-dilution method was used to determine the MIC of the test compounds. Concentrations ranging from 100 to 1,000 μg/ml were used for the study. The minimum inhibitory concentration of MAF and BAF obtained with different microbes were as follows (Table 1).

**Table 1:** Minimum inhibitory concentration of test compounds.

Compound	*C. violaceum*	*P. aeruginosa*
MAF	500 μg/ml	500 μg/ml
BAF	No growth inhibition	No growth inhibition

### 3.2 Effect of sub-inhibitory concentration of the test compounds on violacein production and biofilm formation of *Chromobacterium violaceum*

**Figure 2:**
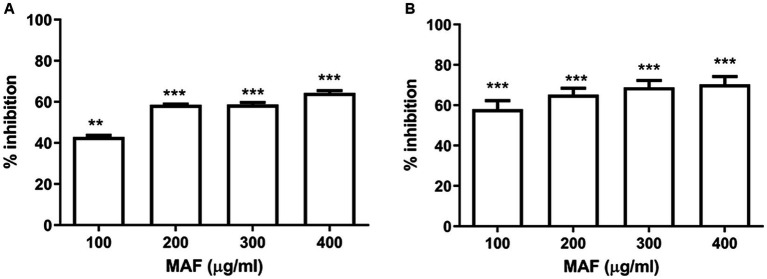
Dose-dependent effect of MAF on violacein production **(A)** and biofilm formation **(B)** of *C. violaceum*. The data represents the average of three independent experiments. The values are expressed as means standard deviation. Dunnett’s test demonstrates significant difference between the tests and the control. Significance at *p < 0.05 vs. control, **p < 0.01 vs. control, ***p < 0.001 vs. control.

Production of purple-colored violacein pigment in *C. violaceum* is regulated by quorum sensing. Violacein production is a noticeable phenotype that facilitates the detection of quorum-sensing inhibitors using this bacterium. The amount of pigment produced by *C. violaceum* 12,472 on exposure to sub-inhibitory concentrations of test compounds were evaluated using the broth dilution method. As the minimum inhibitory concentration of MAF is 500 μg/ml, sub-MIC (i. e., 100–400 μg/ml) was checked for the inhibitory effect of violacein production. In the presence of 400 μg/ml of MAF, a significant reduction in violacein production was observed (64%) (Figure 2A). Conversely, BAF up to the concentration of 500 μg/ml did not cause any inhibitory effect on the growth of *C. violaceum*.

During the growth and multiplication phases, bacteria exist in two forms such as independent planktonic form and as sessile aggregates/biofilms. Sub-MIC concentrations of MAF significantly interfere with biofilm development, resulting in the inhibition of biofilm formation of *C. violaceum* by 64.5% (Figure 2B), whereas BAF did not show any effect on the biofilm formation of *C. violaceum*. These results indicate that MAF possesses both anti-QS and anti-biofilm activity in *C. violaceum*.

### 3.3 Effect of MAF on *Pseudomonas aeruginosa*

#### 3.3.1 Effect of MAF on *Pseudomonas aeruginosa* growth

**Figure 3:**
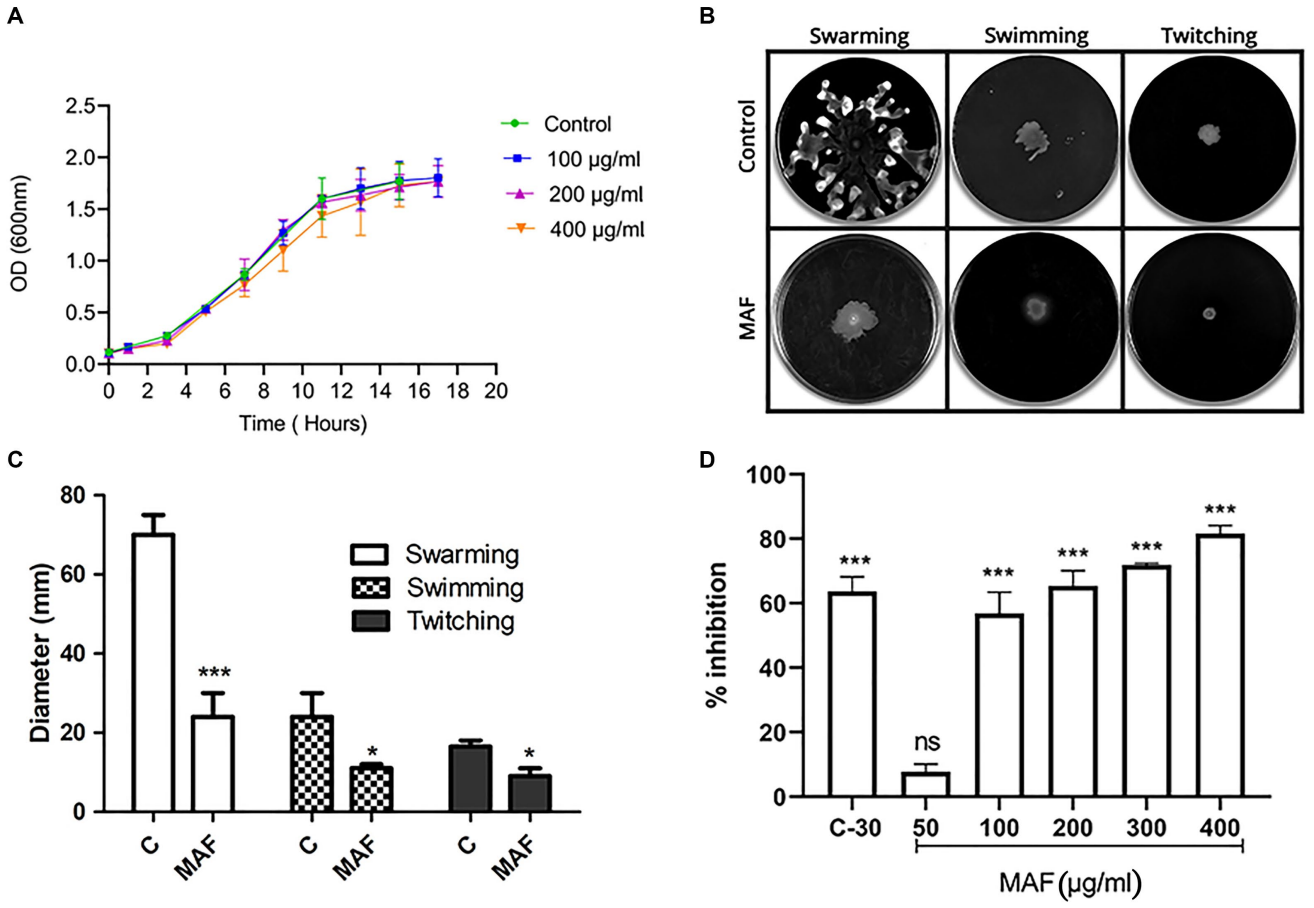
Effect of MAF on growth of *P. aeruginosa* **(A)**. Effect of MAF on swimming, swarming, and twitching motility exhibited by *P. aeruginosa* **(B)**. Motility measured by taking the diameter of bacterial growth in agar media **(C)**. Percentage inhibition of biofilm formation in a dose-dependent manner **(D)**. The data represents the average of three independent experiments. The values are expressed as means standard deviation. Dunnett’s test demonstrates a significant difference between the tests and the control. Significance at *p < 0.05 vs. control, **p < 0.01 vs. control, ***p < 0.001 vs. control.

The growth curve investigation of *P. aeruginosa* PAO1 in the presence of various concentrations of MAF (100, 200, and 400 μg/ ml) demonstrated no inhibitory effect on bacterial cells compared with the untreated control (Figure 3A).

#### 3.3.2 Effect of MAF on motility and biofilm formation of *Pseudomonas aeruginosa*

*Pseudomonas aeruginosa* exhibits various types of motilities which include swimming, swarming, and twitching, all of which are important virulence factors. As observed in Figure 3B, the diameter of the spreading colony decreased in the presence of 400 μg/ml of MAF compared with the control, indicating that MAF considerably inhibited the swarming motility of *P. aeruginosa*.

The swimming motility, which is another key motility exhibited by *P. aeruginosa*, plays a major role in the premature stages of biofilm formation and, like swarming motility, is also regulated by QS (Cai et al., 2020). As shown in Figure 3C, MAF reduced the average diameter of the bacterial growth when compared to the control (29%), demonstrating that it hinders the swimming motility of *P. aeruginosa* PAO1. Furthermore, the twitching motility was also reduced by MAF (27%). These results clearly indicated that MAF affected the initial stages of biofilm formation of *P. aeruginosa* (Figure 3C).

In *P. aeruginosa*, biofilm formation is partially regulated by QS (Yan and Wu, 2019). Multi-layered bacterial cells are entangled in the polysaccharide matrix of the biofilm (Karygianni et al., 2020). Sub-inhibitory concentration of MAF and BAF were assessed for its inhibitory effect on biofilm formation where a dose-dependent decrease in biofilm formation was observed. MAF at a concentration of 400 μg/ml showed significant inhibition of biofilm formation (81%) compared with the Furanone C-30 (63%) (Figure 3D), whereas 400 μg/ml BAF did not show any anti-biofilm activity in *P. aeruginosa*.

#### 3.3.3 MAF inhibited the pyocyanin production and elastase activity of *Pseudomonas aeruginosa*

**Figure 4:**
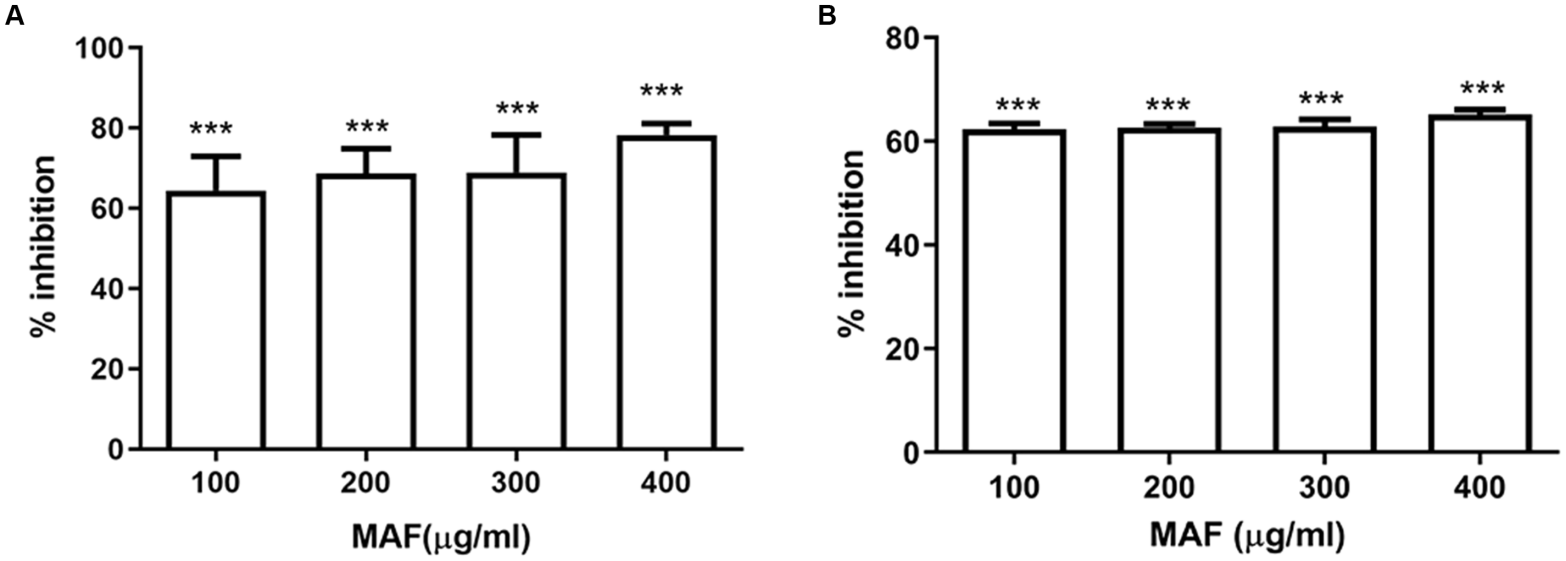
Effect of different doses of MAF on pyocyanin production **(A)** and elastase B activity **(B)** of *P. aeruginosa*. The data shown are mean values of three repeats. The values are expressed as mean standard deviation, *n* = 3. Significance at *p < 0.05 vs. control, **p < 0.01 vs. control, ***p < 0.001 vs. control.

Pyocyanin and pyoverdine are the two main pigments produced by *P. aeruginosa*. The production of pyocyanin, a bluish-green-colored pigment, which plays an important role during pathogenesis, is regulated by QS (Castillo-Juarez et al., 2017). The dose-dependent inhibition of MAF on pyocyanin production is shown in Figure 4. *P. aeruginosa*, upon treatment with 400 μg/ml of MAF, demonstrated a significant inhibition (79%) of pyocyanin production (Figure 4A). Since these observations were obtained at sub-MIC concentrations, they demonstrate that MAF can lower pyocyanin synthesis, while having no detrimental effects on growth of bacteria.

Elastase B is a zinc metalloprotease, which can cause extensive damage to biological tissues (Cigana et al., 2021). The effect of MAF on elastase activity was determined by using Elastin–Congo Red. Elastase activity was significantly reduced when *P. aeruginosa* was treated with MAF at a concentration of 400 μg/ml (55%) (Figure 4B).

#### 3.3.4 Effect of MAF on the expression of QS-controlled genes of PAO1

**Figure 5:**
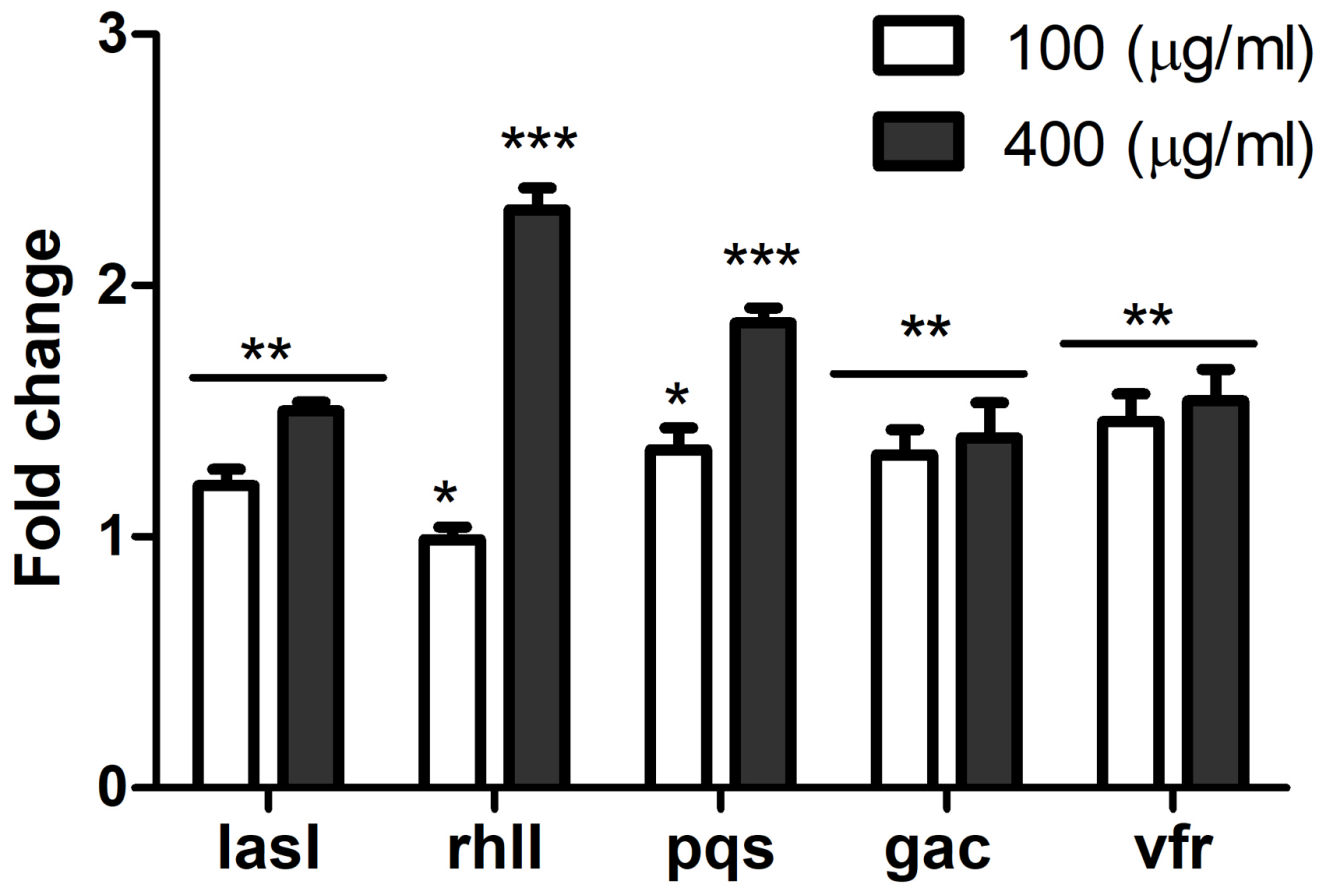
Effect of MAF on expression of QS regulated genes of *P. aeruginosa*. Gene expression studies were conducted in controls and *P. aeruginosa* treated with µg/ml MAF. The data shown are mean values of three repeats. The values are expressed as mean standard deviation, *n* = 3. Significance at *p < 0.05 vs. control, **p < 0.01 vs. control, ***p < 0.001 vs control.

To understand the mechanism underlying the anti-virulent activity, the effect of MAF on the expression of QS circuitry genes was investigated. RNA extracted from *P. aeruginosa* grown under varying concentrations of MAF was subjected to gene expression studies using reverse transcription polymerase chain reaction. Las, Rhl, and PQS are the key QS systems present in *P. aeruginosa*. The present study established that the expression of *lasI*, *rhlI* and *pqsA* genes involved in synthesis of QS molecules were significantly reduced (1.5-fold, 2.3-fold, and 1.85-fold, respectively) when compared with the untreated control (Figure 5). Additionally, compared with the control, the expression of the *gac A* and *vfr* genes, which positively regulate the QS system, was downregulated by 1.3-fold and 1.5-fold, respectively.

### 3.4 Survival of *Caenorhabditis elegans* upon treatment with different concentrations of MAF

**Figure 6:**
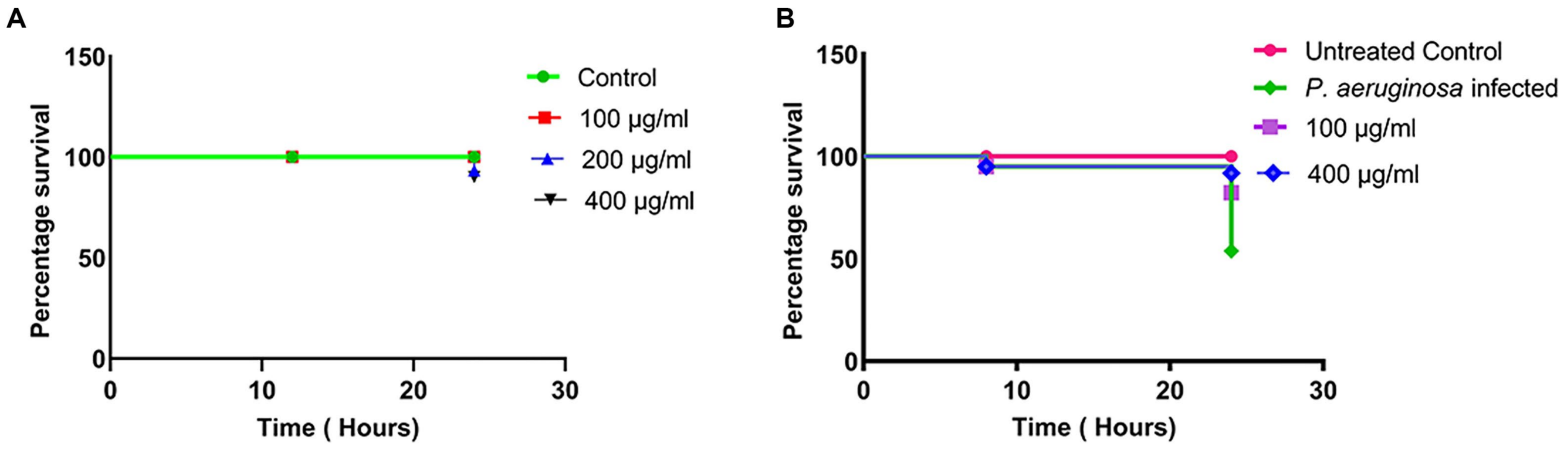
Lethality of *C. elegans* with different doses of MAF **(A)** Survival curves of *C. elegans* infected with *P. aeruginosa* with various treatment groups with MAF **(B)**. The survival rates were subjected to Kaplan–Meier survival analysis to prepare survival curves and the data were compared with untreated worms.

The cytotoxicity of different doses of MAF in *C. elegans*, a widely accepted animal model for toxicity studies, was performed. The results indicate that after treatment with different doses of MAF (100, 200, and 400 μg/ml) there was significant survival (100, 96, and 93% respectively) of the worms even after 24 h, which clearly demonstrated the non-toxicity of the test compounds (Figure 6A).

### 3.5 Survival assay of *Caenorhabditis elegans* upon infection with *Pseudomonas aeruginosa*

Since MAF exhibited significant anti-quorum sensing activity, the anti-infective properties of this compound were tested in a whole animal model. The L1 larval stages of *C. elegans* was infected with *P. aeruginosa* which was previously treated with MAF, and the survival of the worms was assessed at different time points (0, 8, and 24 h). The worms were considered dead when they straightened and became less curved and became inactive without any movement. Upon the addition of test compounds, a dose-dependent increase in the survival of the worms was observed. There was a significant enhancement in the survival of *C. elegans* infected with 400 μg/ml MAF treated *P. aeruginosa* (91%) compared with a 53.8% survival of *C. elegans* infected with untreated *P. aeruginosa*, clearly demonstrating the anti-virulent activity of MAF on *P. aeruginosa* (Figure 6B).

### 3.6 Molecular docking of MAF with *Chromobacterium violaceum* and *Pseudomonas aeruginosa* quorum-sensing receptors

**Figure 7:**
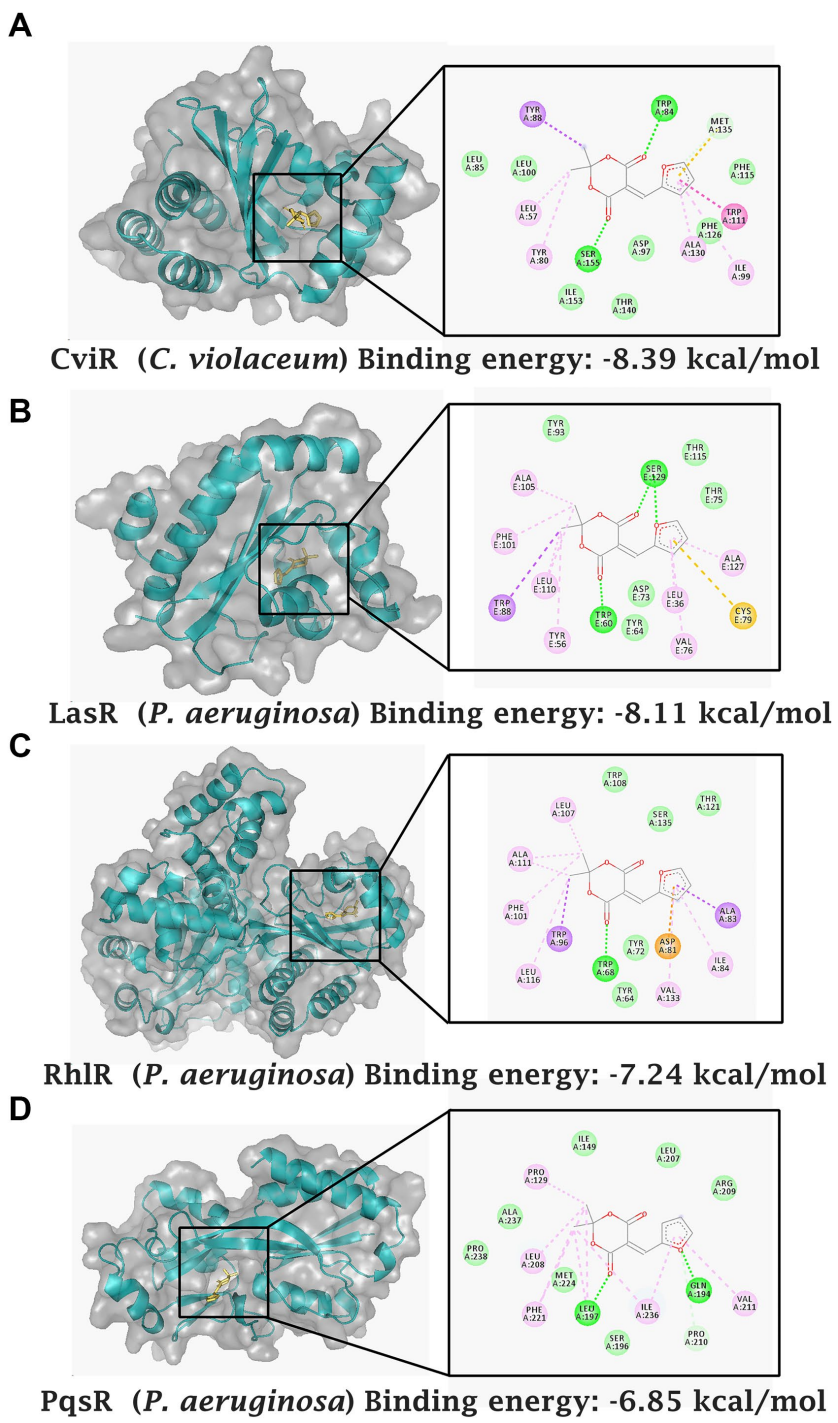
Computational docking analysis of MAF with quorum sensing receptors. CviR receptor of *C. violaceum* **(A)** LasR receptor of *P. aeruginosa* **(B)** RhlR receptor of *P. aeruginosa* **(C)** and PqsR receptor of *P. aeruginosa* **(D)**.

Computational docking studies indicated that the compound was able to bind to CViR of *C. violaceum* with a binding energy of −8.39 kcal/mol. The ligand formed hydrogen bonds with Ser-155 and Trp-84 in the ligand binding domain of CViR of *C. violaceum*. The competitive interaction of MAF with the three important quorum sensing receptors of *P. aeruginosa*, LasR, RhlR, and PqsR were also analyzed. Trp-60 present in the ligand binding domain of LasR formed a hydrogen bond with one of the carbonyl oxygens of the Meldrum’s acid ring, whereas Ser-129 of the LasR depicted two hydrogen bond possibilities which include oxygen of the furan ring and one of the carbonyl oxygens of the Meldrum’s acid. The binding energy was predicted to be −8.11 kcal/mol. The ligand was also able to bind to RhlR with a binding energy of −7.24 kcal/mol forming a single hydrogen bond with Trp-68 in the binding pocket of RhIR. MAF formed hydrogen bonds with Leu-197 and Gln-194 in the co-inducer binding domain of PqsR. The binding energy of interaction was estimated to be −6.85 kcal/mol (Figure 7). Thus, the docking studies predicted the potential of a direct interaction of MAF with the virulence proteins of *C. violaceum* and *P. aeruginosa*.

Computational docking studies were also performed with the natural ligands of different proteins; CviR (N-hexanoyl-L-homoserine lactone C6-HSL), LasR (3-oxo-C12_2_-homoserine lactone, C12-HSL), RhlR (C4-homoserine lactone, C4-HSL), and PqsR (2-heptyl-4- hydroxyquinoline, HHQ). Binding energies obtained are presented in Supplementary Table S1. The molecular interactions (Supplementary Figure S2) of MAF with LasR, RhlR, and PqsR are comparable to their natural ligands suggesting a potential interaction between MAF and the virulence proteins of *C. violaceum* and *P. aeruginosa*.

## 4 Discussion

Widespread and careless use of antimicrobials has significantly contributed to the emergence of drug-resistant microorganisms, thus demanding more efficient novel approaches to tackle this problem. Among the various natural and synthetic compounds that have been studied recently, heterocyclic compounds like furan derivatives appear to be the most promising candidates (Eseyin and Steele, 2015). Furan derivatives such as 5-hydroxymethyl furfural derived from natural products are also reported to exhibit anti-quorum sensing activity (Rajkumari et al., 2019). In this study, we present the investigation of Meldrum’s acid and 1, 3-dimethyl Barbituric acid activated furans (MAF and BAF) as novel quorum sensing inhibitors against different microorganisms.

The production of violacein, the pigment produced by *C. violaceum*, is regulated by QS. Reduction of pigment formation mediated by MAF, provided the primary evidence of the QS inhibitory property of the test compound. Recent reports (Rajkumari et al., 2019) demonstrate the quorum quenching potential of 5-hydroxymethylfurfural, a natural furfural derivative against *P. aeruginosa* and *C. violaceum*. As an opportunistic pathogen, *P. aeruginosa* is highly adaptive and displays significant resistance toward many antimicrobial agents. Using compounds without bactericidal or bacteriostatic effects is a suitable way to tackle this pathogen (Høiby et al., 2010). *P. aeruginosa* has the ability to form intractable biofilms, which are highly resistant to antibiotic treatment. In biofilm, *P. aeruginosa* aggregate together in the matrix, comprising of polysaccharides, proteins, and eDNA, which provides a safe shelter and also helps in the spread of chronic infections (Yin et al., 2022). The sub-inhibitory concentration of MAF significantly reduced the biofilm formation of *P. aeruginosa* compared with the untreated control. The inhibitory effect of MAF on the biofilm formation of *P. aeruginosa* is comparable with the positive control Furanone C-30. Furanone C-30 is reported to inhibit both the LasR and RhlR quorum sensing system of *P. aeruginosa* (Markus et al., 2021). Furanone C-30 downregulated the expression of various virulence genes and genes involved in the production of AHL. Earlier studies established that Furanone compounds efficiently inhibited the initial attachment of planktonic *P. aeruginosa* and reduced rhamnolipid production, which is essential for maintaining biofilm structure and integrity (Proctor et al., 2023). In the present study, Furanone C-30 was used as a known biofilm inhibitor of *P. aeruginosa*. It was observed that MAF at a concentration of 400 μg/ ml significantly reduced (81%) biofilm formation of *P. aeruginosa* compared to the positive control Furanone C-30 which showed 63% biofilm inhibition. In addition to motility, exopolysaccharides and eDNA also contribute to biofilm formation. The different polysaccharides and pili are regulated by the GacA-GacS two-component system. GacA is involved in the positive regulation of the biofilm formation and production of virulence factors such as pyocyanin and lipase (Skariyachan et al., 2020). Gene expression studies demonstrated that sub-MIC concentrations of MAF significantly downregulated the *gac A* gene, which plays a pivotal role in biofilm formation of *P. aeruginosa*. These results demonstrate the high potential of anti-biofilm activity of MAF compared with natural 5-HMF reported by Rajkumari et al.

The different types of motilities exhibited by *P. aeruginosa*, such as swimming, swarming, and twitching, have a significant impact on biofilm formation. Swarming and swimming motility, which are mediated by flagella, have crucial roles in the biofilm formation and further spread of infection in biotic and abiotic surfaces (Saeki et al., 2021). The coordinated translocation of a bacterial population across a surface by swarming motility is considered a major virulence factor of *P. aeruginosa*. Recent studies by Kumar et al. (2021) indicated that swarming motility is regulated by Las and Rhl signaling systems. Our results demonstrated that flagellar dependent swimming and swarming motility is reduced in the presence of MAF. Furthermore, the inhibition of flagellar motility provides robust indication of QS inhibition. Flagellar independent twitching motility of *P. aeruginosa* helps the movement of organisms on moist surfaces. This QS-regulated trait has a vital role in the establishment of infection and biofilm formation. Pili mediated twitching motility is also regulated by Las and Rhl quorum sensing systems (Kumar et al., 2021). The slight difference in the twitching motility with MAF indicates its direct effect on pili function. Further studies will be focused on understanding the molecular mechanism of action of MAF on flagellar and pili proteins.

The secreted virulence factors play a vital role in the pathogenesis of *P. aeruginosa*, which enables the endurance and establishment of infection (Moradali et al., 2017). The major virulence attributes of *P. aeruginosa* include pyocyanin and elastase which play a vital role in the production of reactive oxygen species and damaging the tissues in the host, respectively (Qin et al., 2022). Pyocyanin level and elastase activity were significantly reduced by MAF to 79 and 55%, respectively. This is in agreement with the previous studies where Rajkumari J. et al. reported a considerable reduction in the pyocyanin pigment production and elastase activity in PAO1 up to 65.34 and 36.48%, respectively, in treatment with 5-HMF (Rajkumari et al., 2019). According to our previous study, the elastase activity in PAO1 was significantly decreased with the treatment of clove oil (Haripriyan et al., 2018). These results demonstrated the potential of MAF in interfering with the QS system of *P. aeruginosa*.

Gene expression studies were performed to understand the mechanism of anti-QS activity of MAF. Expression analysis of quorum sensing genes involved in Las, Rhl, and PQS pathways were carried out. The significant reduction in the expression of genes encoding the autoinducers (*lasI and rhlI*) indicate that MAF reduced the formation of signaling molecules - C12 HSL and C4 HSL. Additionally, the expression of the Gac/Rsm system, which positively regulates QS (Sultan et al., 2021), was also downregulated in the presence of MAF. Furthermore, MAF significantly downregulated the expression of *vfr* (virulence factor regulator), which has a crucial role in the transcriptional flow that enhances *P. aeruginosa* pathogenicity (Serate et al., 2011), and the *pqsA* gene, involved in the formation of the precursor of PQS through the kynurenine pathway (Genestet et al., 2014); (YM et al., 2008). These results validate the previous observations of inhibition of elastase activity, pyocyanin, and biofilm formation in the presence of sub-inhibitory concentrations of MAF.

*In silico* analysis demonstrates that MAF acted as a potential competitor for QS-receptors such as CviR of *C. violaceum* and LasR, RhlR, and PqsR receptors of *P. aeruginosa*. However, in *P. aeruginosa*, LasR showed the highest binding affinity towards MAF among the three receptor proteins analyzed. Hence, the computational docking analysis indicated the potential of MAF to form a stable interaction with the binding pockets of CviR of *C. violaceum* and LasR of *P. aeruginosa*. In accordance with our observations, previous studies have also reported potential interactions with 5 hydroxymethyl furfural and LasR in computational docking analysis (Rajkumari et al., 2019). However, we observed that in comparison with HMF, MAF exhibited a higher binding affinity to LasR.

*C. elegans* has been widely employed in several scientific domains, including toxicity studies and as an infection model, due to a variety of beneficial qualities (e.g., short life and reproduction cycle and it being robust, easy, and cheap to maintain huge populations). Our lethality studies revealed that MAF is non-toxic to *C. elegans. C. elegans* is considered an excellent model organism to study the anti-virulent properties of natural and synthetic compounds (Kong et al., 2014). The major contributors of *P. aeruginosa* mediated *C. elegans* mortality are cyanide asphyxiation, which leads to paralysis and further death of the animal (Kumar et al., 2021). Infection of *C. elegans* with MAF treated *P. aeruginosa* established considerable protecting effect on the worm’s mortality. Earlier reports from our lab established similar types of protective properties of clove bud oil (Haripriyan et al., 2018).

## 5 Conclusion

Meldrum’s acid activated furan (MAF) and 1,3-dimethyl barbituric acid activated furan (BAF) were successfully synthesized. The quorum quenching properties of the MAF were established in *C. violaceum* and *P. aeruginosa*. This promising approach could be explored to reduce the emergence of drug resistant pathogens. Gene expression studies and *in-silico* analysis confirmed the anti-virulent potential of MAF in *P. aeruginosa*. MAF has the potential to be used as an anti-virulence drug since it increases the survival of *C. elegans* by reducing the pathogenicity of *P. aeruginosa*. This report is the first report of its kind using synthetic furan compounds in combating critical priority pathogen *P. aeruginosa*. Future studies are being undertaken in *P. aeruginosa* to establish the detailed mechanism of action of MAF on biofilm formation, biofilm dispersion, and regulation. Once the mechanisms of action of MAF in different organisms is elucidated, the insights derived from this study can be employed to generate a library of activated furan compounds as potential anti-quorum sensing agents.

## Data availability statement

The original contributions presented in the study are included in the article/Supplementary material, further inquiries can be directed to the corresponding authors.

## Author contributions

AS: Formal analysis, Investigation, Methodology, Validation, Writing – review & editing. JV: Formal analysis, Investigation, Methodology, Writing – original draft. ARa: Formal analysis, Investigation, Writing – review & editing. ARe: Formal analysis, Investigation, Methodology, Writing – review & editing. DR: Formal analysis, Investigation, Methodology, Writing – review & editing. DP: Formal analysis, Investigation, Methodology, Writing – review & editing. MV: Investigation, Methodology, Writing – review & editing. JV: Formal analysis, Investigation, Methodology, Validation, Writing – review & editing. RD: Methodology, Project administration, Resources, Supervision, Writing – review & editing. SH: Project administration, Resources, Supervision, Writing – review & editing. SS: Formal analysis, Methodology, Validation, Writing – review & editing. PC: Conceptualization, Resources, Supervision, Writing – review & editing. GK: Conceptualization, Data curation, Writing – review & editing. SV: Conceptualization, Funding acquisition, Writing – original draft, Writing – review & editing. JH: Conceptualization, Data curation, Funding acquisition, Writing – original draft, Writing – review & editing.
