# Supplementary material for: Mitigation of quorum sensing mediated virulence factors of Pseudomonas aeruginosa: the role of Meldrum’s acid activated furan
Source: Front Microbiol. 2024 Jan 3;14:1272240. doi: 10.3389/fmicb.2023.1272240 (PMC10791761; doi:10.3389/fmicb.2023.1272240)
Supplement: Supplementary file 1 [file Data_Sheet_1.pdf]

Supplementary Material

Mitigation of Quorum Sensing Mediated Virulence factors of *P. aeruginosa*: Role of Meldrum's Acid Activated Furan

Ajmal Sadik [1], Jithin P. Viswaswar [1], Ambili Rajamoney [1], Anjali Rekha [1], Darsana M. Raj [1], Deepthi Prakashan [1], Mydhili Vasudevan [1], J. S. Visakh [1], Dhannya Renuka [1], Sreetha Hely [1], Sanu Korumadathil Shaji [1†], Prakash R. Chandran [2], Geetha Kumar [1], Sobha Vijayan Nair [1*] and Jayalekshmi Haripriyan [1*]

[1] School of Biotechnology, Amrita Vishwa Vidyapeetham, Amritapuri, Kerala, India,

[2] Department of Chemistry, Mannam Memorial N.S.S. College, Kottiyam, Kerala, India

*Corresponding author/s:

Jayalekshmi Haripriyan, jayalekshmih@am.amrita.edu

Sobha V. Nair, sobhavn@am.amrita.edu

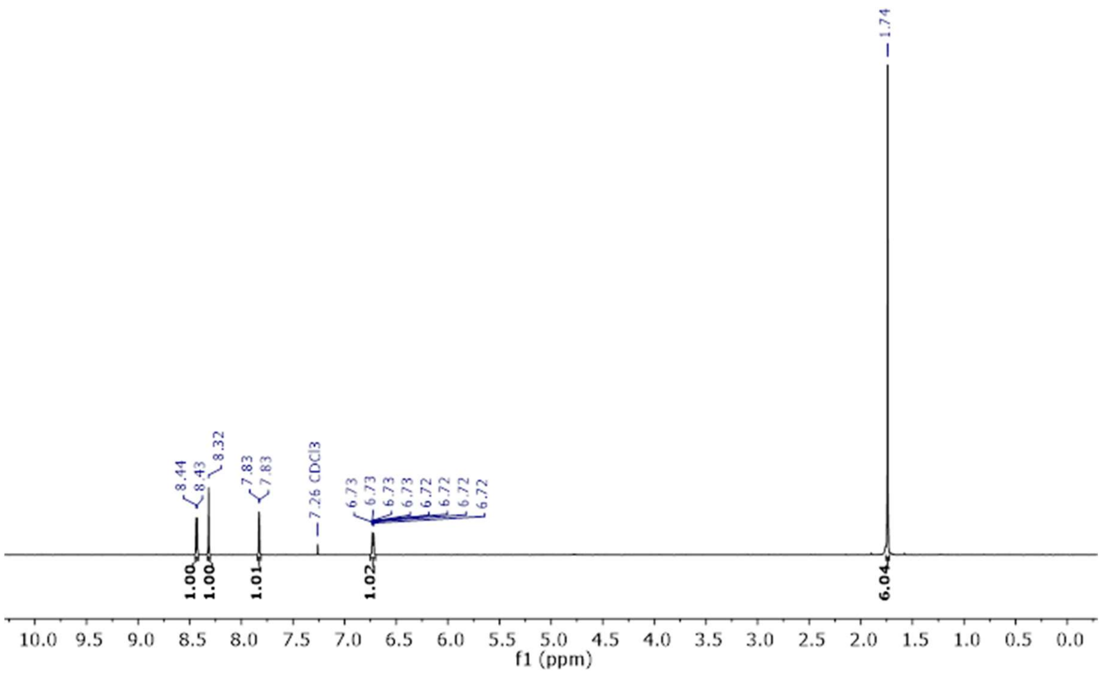

Figure S1(A): ^{1}HNMR of Meldrum's acid Activated Furan (MAF)

^{1}H NMR (400 MHz, Chloroform-*d*) δ 8.43 (d, *J* = 3.9 Hz, 1H), 8.32 (s, 1H), 7.83 (d, *J* = 1.3 Hz, 1H), 6.73 (ddd, *J* = 3.8, 1.6, 0.7 Hz, 1H), 1.74 (s, 6H).

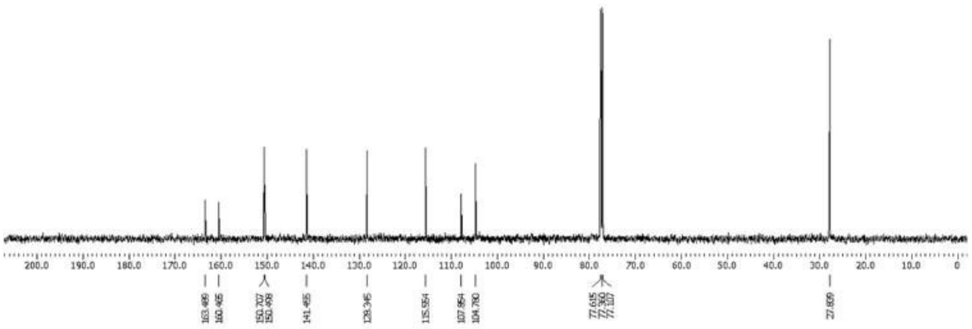

Figure S1(B): ^{13}CNMR of Meldrum's acid Activated Furan (MAF)

^{13}C NMR (125 MHz, $CDCl_3$) δ163.5 (C=O), 160.5 (C=O), 150.7 (C), 150.5 (CH), 141.5(CH), 128.3(C), 115.8 (CH), 107.9 (CH), 104.8 (C), 27.8 (2xCH_3)

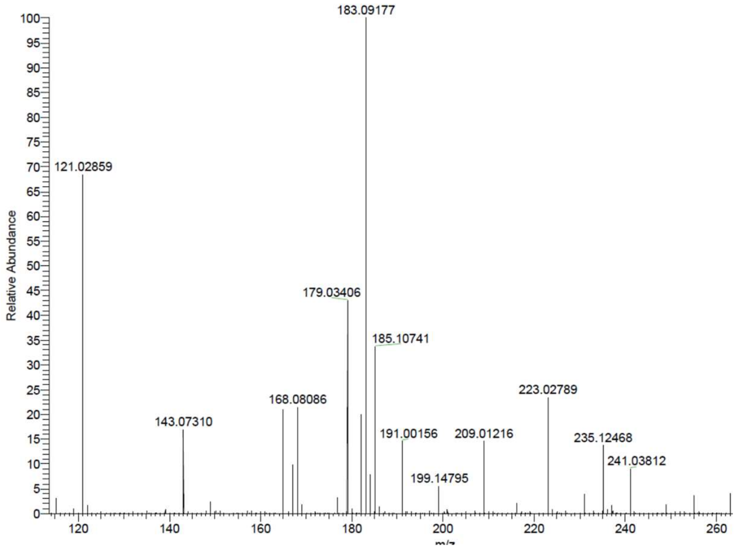

Figure S1(C): Mass spectra of Meldrum's acid Activated Furan (MAF)
MS (ESI^+) m/z 223.0278 $[(M+H)^+$

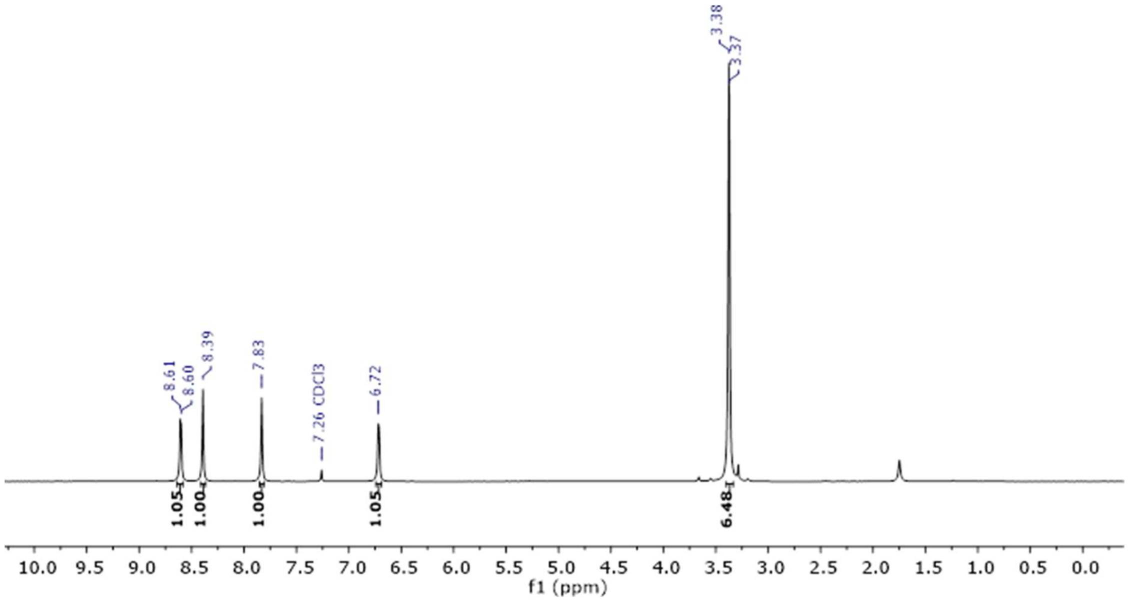

Figure S1(D): ^{1}HNMR of 1,3-dimethyl Barbituric acid Activated Furan (BAF)

^{1}H NMR (400 MHz, Chloroform-*d*) δ 8.60 (d, J = 3.4 Hz, 1H), 8.39 (s, 1H), 7.83 (s, 1H), 6.72 (s, 1H), 3.37 (d, J = 2.4 Hz, 6H).

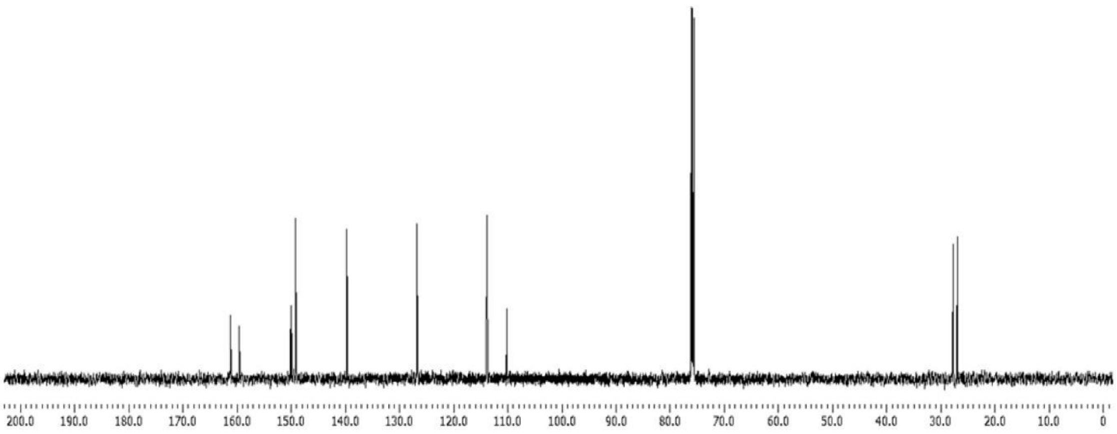

Figure S1(E): ^{13}CNMR of 1,3-dimethyl Barbituric acid Activated Furan (BAF)

^{13}C NMR (125 MHz, $CDCl_3$) δ161 (C=O), 159 (C=O), 150 (C=O), 149 (C), 139 (2xCH), 127.5 (C), 113.5 (2xCH), 28 (CH_3),27 (CH_3)

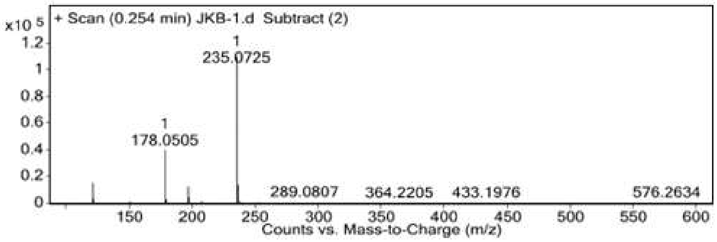

Figure S1(F): Mass spectra of 1,3-dimethyl Barbituric acid Activated Furan (BAF)
MS (ESI^+) m/z 235.0725 $[(M+H)^+$

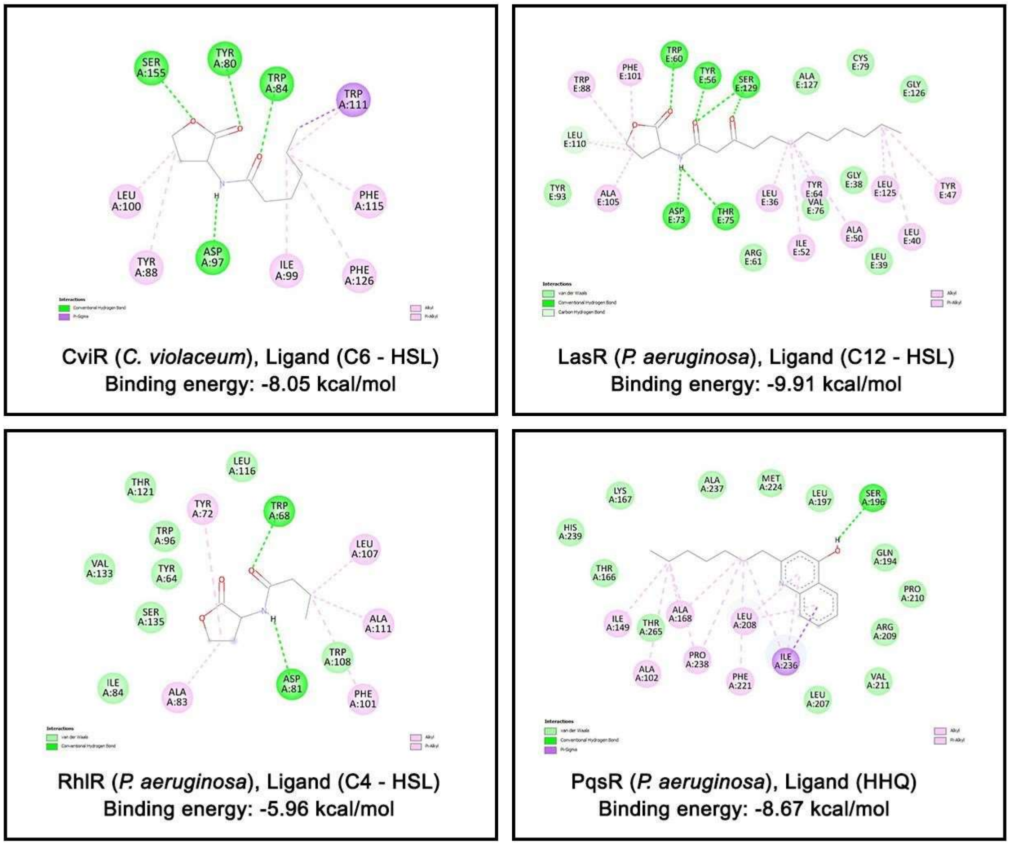

Figure S2: Computational docking analysis of natural ligands with QS receptors

Protein	Ligand	Binding Energy (kcal/mol)
CViR	N-hexanoyl-L-homoserine lactone (C6-HSL)	-8.05
	MAF	-8.39
LasR	3- oxo-C12-homoserine lactone (C12-HSL)	-9.91
	MAF	-8.11
PqsR	C4-homoserine lactone (C4-HSL)	-5.96
	MAF	-7.24
RhlR	2-heptyl-4-hydroxyquinoline (HHQ)	-8.67
	MAF	-6.85

Table ST1: Binding energies of virulence proteins with their natural ligands and MAF
